# Frequency-converted dilute nitride laser diodes for mobile display applications

**DOI:** 10.1186/1556-276X-9-82

**Published:** 2014-02-17

**Authors:** Janne Konttinen, Ville-Markus Korpijärvi

**Affiliations:** 1 EpiCrystals Oy, Hermiankatu 12 E 2, Tampere 33720, Finland; 2 Optoelectronics Research Centre, Tampere University of Technology, Korkeakoulunkatu 3, Tampere 33720, Finland

**Keywords:** Semiconductor laser, Second harmonic generation, Visible, III-V semiconductors, Dilute nitride, GaInNAs, Mobile display, Semiconductor lasers, Laser diodes 42.55.Px, Second harmonic generation, 42.65.Ky

## Abstract

We demonstrate a 1240-nm GaInNAs multi-quantum well laser diode with an integrated saturable electro-absorber whose wavelength is converted to 620 nm. For conversion, we used a MgO:LN nonlinear waveguide crystal with an integrated Bragg grating in direct coupling configuration. Broadened visible spectral width and reduced speckle as well as a high extinction ratio between the below and above threshold powers were observed in passively triggered pulsed operation with smooth direct current modulation characteristics. The demonstration opens a new avenue for developing 620-nm semiconductor lasers required for emerging projection applications.

## Background

Red laser light sources emitting in the wavelength range of 610 to 620 nm are particularly interesting for mobile display applications due to increased luminous efficacy and higher achievable brightness within eye-safety regulations [1]. Unfortunately, this wavelength range is difficult to achieve by using traditional GaInP/AlGaInP red laser diodes (LDs) [2]. Another well-known drawback of GaInP/AlGaInP diodes is the reduction of characteristic temperature of threshold current (*T*_0_) with wavelength. High *T*_0_ values have been demonstrated with red laser diodes emitting at wavelengths above 650 nm [3], while shorter wavelength diodes suffer from poor temperature characteristics [4]. These features render impossible the use of standard AlGaInP laser diodes in embedded projection displays, where large operating temperature range is typically required.

Frequency conversion of infrared laser emission is an attractive solution for the generation of short-wavelength red light [5]. While GaInAs quantum well (QW) emission wavelength is practically limited to approximately 1200 nm [6], by using dilute nitride GaInNAs QWs with a tiny fraction of nitrogen added to the highly strained GaInAs, the emission wavelength can be extended to 1220-1240 nm for high luminosity red light generation at 610 to 620 nm by frequency conversion [5]. In addition, excellent temperature characteristics and high power operation have been demonstrated with GaInNAs laser diodes in this wavelength range [7].

## Methods

**Figure 1:**
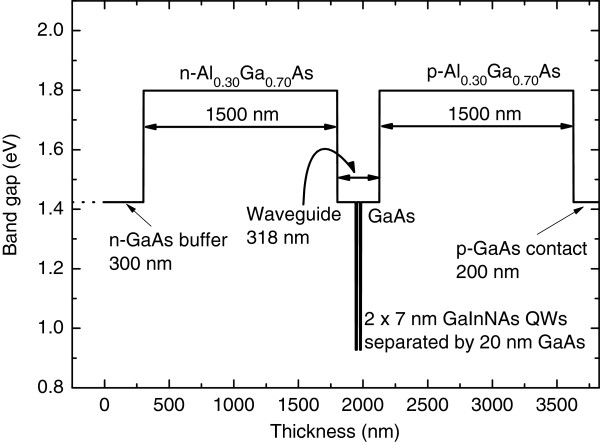
Band gap profile and layer thicknesses of the semiconductor heterostructure of the 1240-nm GaInNAs laser.

**Figure 2:**
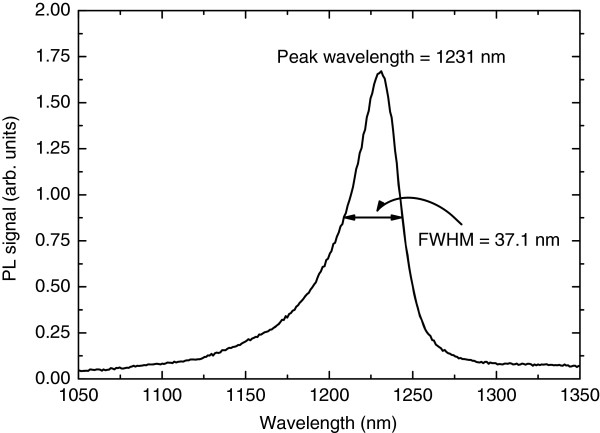
Room-temperature PL emission measured from the 1240-nm GaInNAs/GaAs laser wafer.

The GaInNAs/GaAs semiconductor heterostructure was grown on an n-GaAs (100) substrate by Veeco (Plainview, NY, USA) GEN20 molecular beam epitaxy (MBE) reactor with a radio frequency plasma source for nitrogen, a valved cracker for arsenic, and normal effusion cells for the group-III materials and dopants. Silicon and beryllium were used as n- and p-type dopants. The active region of the laser structure consisted of two 7-nm thick GaInNAs QWs separated by a 20-nm GaAs layer. The Ga_1 - *x*_In_*x*_N_*y*_As_1 - *y*_ QWs had the nominal indium and nitrogen compositions of *x* = 33.6% and *y* = 0.6%, respectively. This double-QW structure was embedded in GaAs whose thickness was 142 nm on both sides of the structure. The undoped waveguide structure was surrounded by 1.5-μm thick n-Al_0.30_Ga_0.70_As on the substrate side and 1.5 μm p-Al_0.30_Ga_0.70_As on the top side. On top of the p-AlGaAs cladding, a p-GaAs contact layer was grown to finalize the structure. Figure 1 shows the band gap profile of the structure and summarizes the layer thicknesses. Strong room-temperature photoluminescence (PL) emission measured from this structure peaked at 1231 nm, as shown in Figure 2. Two heterostructures, comprising one or two QWs, were considered for the frequency-doubled 620-nm laser demonstration. The single-QW and double-QW structures were compared as broad-area ridge-waveguide (RWG) lasers in pulsed current mode. The double-QW structure was opted because it showed only slightly higher threshold current as compared with the single-QW structure (adding the second QW to the test structure increased the threshold current density from 500 to 610 A/cm^2^), and double-QW lasers are known to be less temperature sensitive, i.e., to have larger *T*_0_[8], which is important for the targeted application. The difference between the slope efficiency values of the single-QW and double-QW structures was negligible.

The processed laser chips employed a single transverse mode RWG process with ridge width of 3.5 μm and cavity length of 1250 μm. The laser diode further comprised an 85-μm reverse-biased saturable electro-absorber section to passively trigger short pulses for enhancing frequency conversion efficiency in the nonlinear waveguide. The front and rear facets of the laser diode were AR/HR coated with reflectivities of <1% and >95% at 1240 nm, respectively.

A nonlinear waveguide crystal made of MgO-doped LiNbO_3_ with high nonlinear coefficient was used for frequency doubling to visible wavelengths. The crystal had a surface Bragg grating implemented near the output end of the waveguide. The function of the surface Bragg grating is to provide self-seeding to frequency lock the IR laser diode in order to maintain sufficient spectral overlap with acceptance spectrum of quasi-phase-matched (QPM) grating over an extended temperature range.

## Results and discussion

### Free-running performance

**Figure 3:**
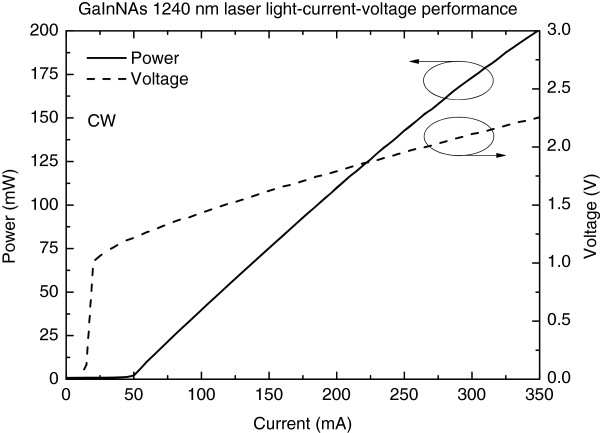
Continuous wave performance of a single-mode 1240-nm GaInNAs laser diode.

**Figure 4:**
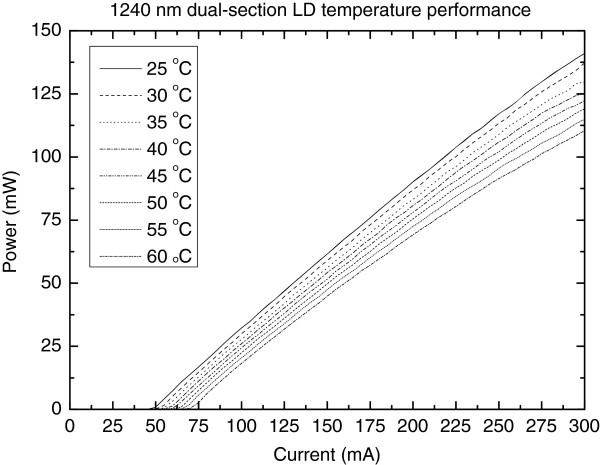
Continuous wave performance of a single-mode 1240-nm GaInNAs laser diode at elevated temperatures.

**Figure 5:**
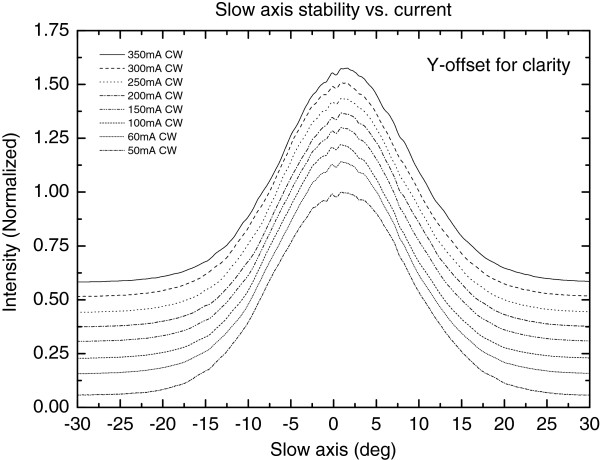
Lateral far-field stability vs. current in continuous wave mode at room temperature.

In free-running mode with the absorber section unbiased, the 1240-nm RWG laser diode exhibited an average slope efficiency of approximately 0.7 W/A and smooth *L*-*I* characteristics at 25°C as shown in Figure 3. The temperature performance was investigated in continuous wave (CW) mode (i.e. the absorber section forward biased by a contact to gain section). Kink-free operation up to 300 mA was demonstrated over the temperature range from 25°C to 60°C, as shown in Figure 4. The corresponding characteristic temperature (*T*_0_) was 97 K for the low front-facet reflectivity-coated free-running LDs (see Figure 4). As it can be seen in Figure 5, the lateral far field exhibited stable single-mode operation up to 350 mA with no evidence of beam steering. The beam opening angles (FWHM) were 40° and 17° for fast and slow axes, respectively. Comparing the measured threshold current and *T*_0_ values with the values of related red AlGaInP-based laser diodes is difficult, because these lasers can hardly reach lasing at 620 nm at normal temperature and pressure. Commercial single-transverse-mode RWG laser diode operating at longer wavelengths (633 nm) [9] has a threshold current of about 60 mA at 25°C, which is identical to the value of the GaInNAs laser reported here. Based on the data available on the datasheet [9], the *T*_0_ of this commercial laser diode is estimated to be 89 K, which comes close to the value reported here for the GaInNAs laser. However, the *T*_0_ value of free-running GaInNAs diode is suppressed due to the low front-facet reflectivity [10] and can thus be improved by providing the wavelength locking optical feedback from Bragg grating in nonlinear waveguide [11]. In addition, it is known that the performance of AlGaInP-based laser diodes, especially their *T*_0_ values, deteriorate strongly as the wavelength is decreased towards 620 nm [4,12,13].

### Frequency conversion

**Figure 6:**
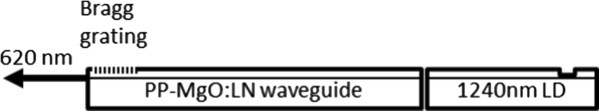
Coupling configuration of passively pulsed frequency-converted 620-nm laser.

The passively pulsed frequency-converted 620-nm laser configuration is shown in Figure 6. The 1240-nm infrared emission from the GaInNAs laser diode is directly coupled to MgO:LN waveguide for single-pass frequency conversion. The surface Bragg grating is implemented near the output end of the nonlinear waveguide, while the reverse-biased saturable absorber is located near the highly reflective back facet of the laser diode. Both facets of the nonlinear waveguide, as well as the output facet of the laser diode, are AR-coated to suppress interface reflections.

**Figure 7:**
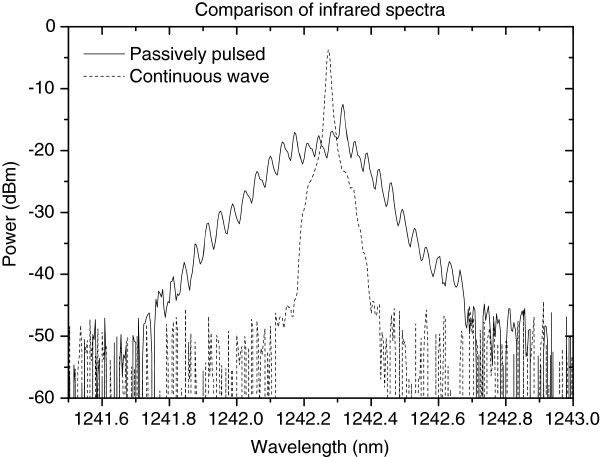
Comparison of grating-locked infrared spectra under continuous wave (dashed line) and pulsed (solid line) operating modes.

**Figure 8:**
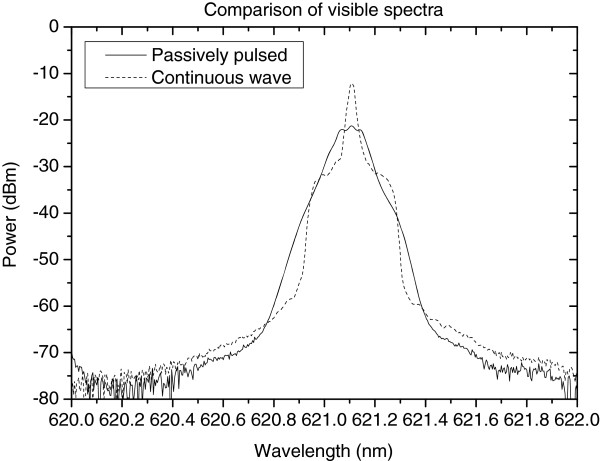
Comparison of grating-locked visible spectra under continuous wave (dashed line) and pulsed (solid line) operating modes.

Successful wavelength locking and passively pulsed operation (with absorber reverse biased) are achieved with the direct coupling configuration between the GaInNAs laser diode and MgO:LN waveguide. The infrared and visible spectra were recorded using Yokogawa AQ6373 optical spectrum analyzer (Tokyo, Japan) with extended wavelength range. Compared with the CW mode, the infrared (Figure 7) and visible spectra (Figure 8) are broadened when the absorber section was biased with 0.4- to 1.5-V reverse-bias voltage triggering passively pulsed mode. A considerable reduction in the speckle visibility is observed under pulsed mode when compared with continuous wave operation. This observation is supported by the measured broadening of the visible spectrum.

**Figure 9:**
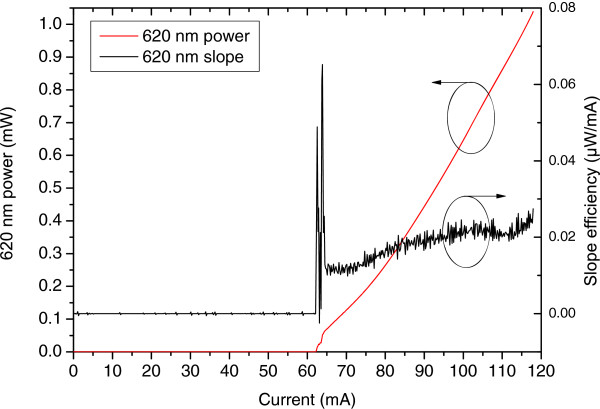
**Frequency-converted 620-nm *L*-*I *** **performance under passively pulsed mode.**

The *L*-*I*-*V* performance under the passively pulsed reverse-biased mode was investigated using 0.2-mA current resolution in the visible output power range of 0 to 1 mW, as targeted for near-to-eye display applications. The lasing threshold was 63 mA under 0.4-V reverse bias. Above the lasing threshold, the visible light output represented smooth, slightly non-linear *L*-*I* curve within the targeted operating power range. The results are summarized in Figure 9.

**Figure 10:**
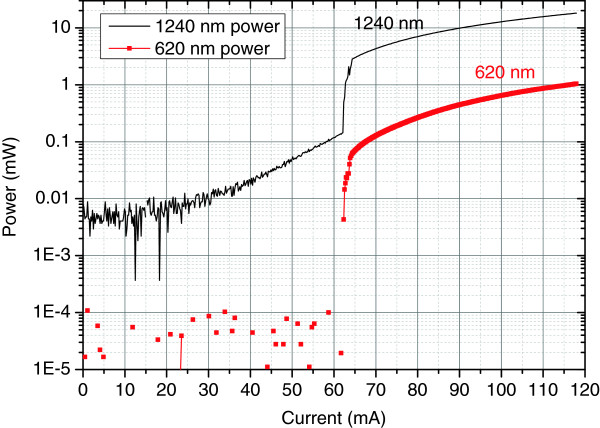
Comparison of frequency-converted 620-nm and infrared 1240-nm output.

**Figure 11:**
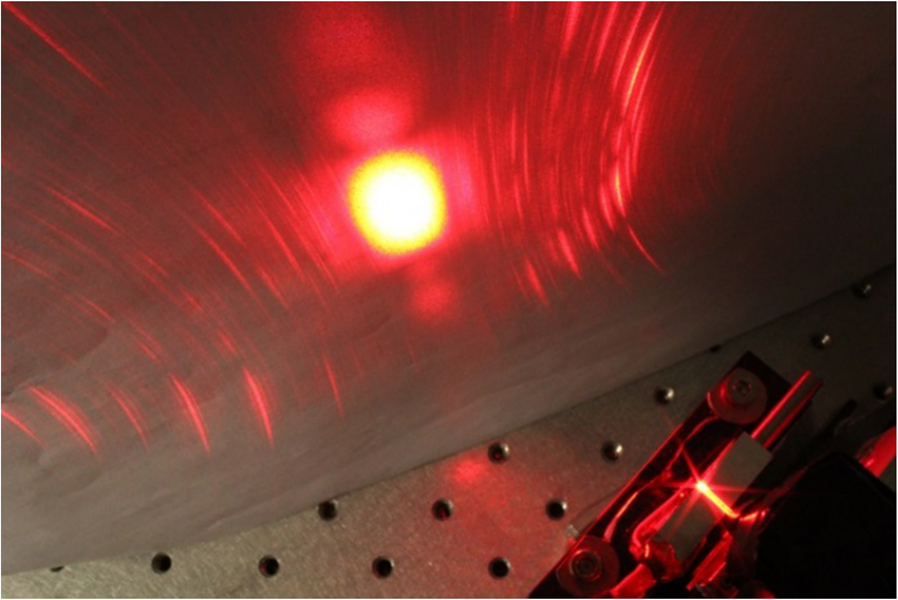
**Projected 620-nm output beam of the GaInNAs laser diode.** MgO:LiNbO_3_ nonlinear waveguide crystal was used for single-pass frequency conversion from 1240 to 620 nm.

The exceptional feature of the 620-nm frequency converted visible light source with ‘no visible light below lasing threshold’ is presented in Figure 10, where the emitted infrared light and visible light are shown with logarithmic *Y*-axis scale. Below the lasing threshold, there is spontaneous infrared emission up to 150 μW, while the visible light emission remained below the detector responsivity limit. When considering applications requiring high contrast ratio, such as near-to-eye and head-up displays, this greatly enhanced extinction ratio is expected to be of particular importance. The projected output beam of the 620-nm laser is presented in Figure 11.

## Conclusions

A transversally single-mode frequency-converted GaInNAs-based 620-nm laser diode is demonstrated with high single pass conversion efficiency and extinction ratio. Further improvements of threshold current and conversion efficiency are expected by optimizing the laser diode manufacturing process and optical coupling configuration.

## Abbreviations

AR: anti-reflection; CW: continuous wave; FWHM: full-width at half maximum; HR: high reflection; LD: laser diode; L-I-V: light-current-voltage; LN: lithium niobate (LiNbO_3_); MBE: molecular beam epitaxy; PL: photoluminescence; QPM: quasi-phase match; QW: quantum well; RWG: ridge waveguide; T0: characteristic temperature of threshold current.

## Competing interests

The authors declared that they have no competing interests.

## Authors’ contributions

JK carried out the laser performance characterization and writing the manuscript. VMK carried out the molecular beam epitaxy and participated in designing the semiconductor structure and writing the manuscript. Both authors read and approved the final manuscript.

## Authors’ information

JK is CTO at EpiCrystals. VMK is a PhD student at the Optoelectronics Research Centre of Tampere University of Technology.

## References

[B1] Buckley E Detailed eye-safety analysis of laser-based scanned-beam projection systems J Displ Technol 2012 9 166 173

[B2] Bohdan R Bercha A Trzeciakowski W Dybała F Piechal B Sanayeh MB Reufer M Brick P Yellow AlGaInP/InGaP laser diodes achieved by pressure and temperature tuning J Appl Phys 2008 9 063105 10.1063/1.2978359

[B3] Onishi T Inoue K Onozawa K Takayama T Yuri M High-power and high-temperature operation of Mg-doped AlGaInP-based red laser diodes IEEE J Quant Electron 2004 9 1634 1638

[B4] Qiu B McDougall S Yanson D Marsh J Analysis of thermal performance of InGaP/InGaAlP quantum wells for high-power red laser diodes Opt Quant Electron 2008 9 1149 1154 10.1007/s11082-009-9314-1

[B5] Härkönen A Rautiainen J Guina M Konttinen J Tuomisto P Orsila L Pessa M Okhotnikov OG High power frequency doubled GaInNAs semiconductor disk laser emitting at 615 nm Opt Express 2007 9 3224 3229 10.1364/OE.15.003224 19532562

[B6] Nakahara K Adachi K Kasai J Kitatani T Aoki M IEEE High-performance GaInNAs-TQW edge emitting lasers20th International Semiconductor Laser Conference: September 17–21 2006; Kohala Coast, HI, USA 2006 Piscataway: IEEE 161 162

[B7] Bisping D Pucicki D Hofling S Habermann S Ewert D Fischer M Koeth J Forchel A High-temperature high-power operation of GaInNAs laser diodes in the 1220–1240-nm wavelength range IEEE Photon Technol Lett 2008 9 1766 1768

[B8] Tansu N Mawst LJ Current injection efficiency of InGaAsN quantum-well lasers J Appl Phys 2005 9 054502 10.1063/1.1852697

[B9] Oclaro Data Sheet HL63163DG AlGaInP Laser Diode, HL63163DG Rev1 http://www.oclaro.com/datasheets/OCDE_HL63163DG_Rev_1.pdf

[B10] Lin C-C Liu K-S Wu M-C Ko S-C Wang W-H Facet-coating effects on the 1.3-μm strained multiple-quantum-well AlGaInAs/InP laser diodes Jpn J Appl Phys 1998 9 6399 6402 10.1143/JJAP.37.6399

[B11] Pliska T Arlt S Matuschek N Schmidt B Mohrdiek S Harder C IEEE High power wavelength stabilized 980 nm pump laser modules operating over a temperature range of 135 K14th Annual Meeting of the IEEE Lasers and Electro-Optics Society (LEOS): November 12–13 2001; San Diego, CA, USA. Volume 1 2001 Piscataway: IEEE 139 140

[B12] Nishida T Shimada N Ogawa T Miyashita M Yagi T Zediker MS Short wavelength limitation in high power AlGaInP laser diodes Proceedings of SPIE: High-Power Diode Laser Technology and Applications IX. Volume 7918 2011 Bellingham: SPIE 791811 791811–7

[B13] Blume G Nedow O Feise D Pohl J Paschke K Monolithic 626 nm single-mode AlGaInP DBR diode laser Opt Express 2013 9 21677 21684 10.1364/OE.21.021677 24104041

